# Outcome of humanitarian patients with late complete repair of tetralogy of Fallot: A 13-year long single-center experience

**DOI:** 10.1016/j.ijcchd.2022.100414

**Published:** 2022-08-10

**Authors:** Damien Schaffner, Guillaume Maitre, Sebastiano A.G. Lava, Yann Boegli, Mirko Dolci, Raymond Pfister, Nicole Sekarski, Perez Marie-Hélène, Stefano Di Bernardo

**Affiliations:** a Pediatric Cardiology Unit, Women Mother and Child Department, Lausanne University Hospital, Switzerland; b Intensive Care Unit, Women Mother and Child Department, Lausanne University Hospital, Switzerland; c Pediatric Anesthesiologic Unit, Anesthesiologic Department, Lausanne University Hospital, Switzerland; d Cardiac Surgery Department, Lausanne University Hospital, Switzerland

**Keywords:** Tetralogy of Fallot, Late complete surgical repair, Humanitarian patients, Pulmonary valve-sparing repair, Pulmonary valve annulus, Postoperative outcome

## Abstract

**Background:**

Surgical repair of tetralogy of Fallot is usually performed between 3 and 6 months of age with pulmonary valve-sparing repair promoted for the best long-term result. Through a humanitarian program from developing countries, late complete surgical repair of tetralogy of Fallot has been performed at our institution for many years.

**Methods:**

Retrospective analysis of pre- and perioperative data, as well as 30-days outcome of patients older than one year with a confirmed diagnosis of tetralogy of Fallot who had a complete surgical repair between 2005 and 2018 at our institution.

**Results:**

One hundred sixty-five patients were included with a median age of 4.5 years [3.0–6.3], median weight of 13.5 kg [10.9 to 16.5], median transcutaneous oximetry of 78% [70 to 85] and median pulmonary valve annulus Z-score of −1.8 [-3.4 to −0.8]. There was no early surgical mortality. By multivariate analysis, only severe right ventricular hypertrophy, severe right ventricle outflow tract obstruction, and hypoplasia of the main pulmonary artery were independent predictors of failure to preserve the pulmonary annulus at surgical repair.

**Conclusions:**

Late complete surgical repair of tetralogy of Fallot has low mortality and morbidity even when pulmonary valve-sparing repair cannot be successfully performed. The preservation of the pulmonary valve function is significantly associated with shorter ventilation time, ICU and hospital lengths of stay. In the analyzed group of patients, a pulmonary valve-sparing repair cannot be predicted exclusively based on the dimension of the pulmonary valve annulus.

## Abbreviations list

BMI: Body mass index
CPB: Cardiopulmonary bypass ECMO Extracorporeal membrane oxygenation
ECMO: Extracorporeal membrane oxygenation
LOS: Length of stay
LPA: Left pulmonary artery
LVPWd: Left ventricle posterior wall thickness in diastole
MPA: Main pulmonary artery
PVA: Pulmonary valve annulus
PVSR: Pulmonary valve-sparing repair
RPA: Right pulmonary artery
RVAWd: Right ventricle anterior wall thickness in diastole
RVOT: Right ventricular outflow tract
RVOTO: Right ventricular outflow tract obstruction
RV-PA: Right ventricle to pulmonary artery conduit
ToF: Tetralogy of Fallot
TP: Transannular patch
VSD: Ventricular septal defect
WHO: World Health Organization

## 1 Background

Tetralogy of Fallot (ToF) is the most common cyanotic congenital heart disease. It is embryologically characterized by anterosuperior deviation of the conal septum creating a spectrum of right ventricular outflow tract obstruction (RVOTO) and a ventricular septal defect (VSD). The degree of RVOTO correlates with the level of cyanosis of the patient.

Since Lillehei in 1954, a one-time complete surgical repair is the worldwide-accepted best therapeutic option [1]. Nowadays, it is uniformly recommended that the surgical repair should be realized between the age of 3–6 months to allow optimal surgical correction, minimize morbidity/mortality and decrease the duration of hypoxemia [[2], [3], [4], [5]]. Usually, surgical mortality is low at approximately 1% [6]. A palliative-shunt procedure or stenting of the right ventricular outflow tract (RVOT) can be performed earlier in the presence of unfavourable preoperative factors (severe RVOTO, small weight, preterm), and complete repair is then postponed. Currently, pulmonary valve-sparing repair (PVSR) is promoted for the best long-term result and to reduce the occurrence of free pulmonary valve regurgitation [7,8].

Our center's partnership with humanitarian non-governmental organizations allows many children from developing countries (Eastern Europe, North and Subsaharan Africa) to be transferred to our institution for surgical repair of their congenital heart defect. Most children have a late diagnosis, and only a minority can access the palliative-shunt procedure in their country. Late surgical repair exposes the child to longer consequences of ToF: severe right ventricular hypertrophy; hypoplasia of the pulmonary vascular bed; severe cyanosis and hypoxic spells. The long-standing hypoxemia leads to polycythemia, cyanotic nephropathy, altered myoarchitecture [9], systolic and diastolic myocardial dysfunction [10], and neurological developmental impairment [11,12]. Finally, these children are prone to malnutrition due to their social conditions and cardiopathy, leading to increased perioperative risks [13,14].

The current study aimed to describe the preoperative anatomical variables in this population treated with Western Countries' surgical and medical standards and analyze the outcomes and the feasibility of PVSR. Secondarily, we analyzed preoperative parameters and related them to the type of surgical repair and their specific outcomes.

## 2 Material and methods

Single-center retrospective observational study, which received Institutional Ethics approval.

### 2.1 Inclusion criteria

Patients with a confirmed diagnosis of ToF, older than one year, referred through a humanitarian organization, who had a complete surgical repair between 2005 and 2018 at the Lausanne University Hospital (CHUV), Lausanne, Switzerland were included.

### 2.2 Exclusion criteria

Children under 12 months at surgical repair or undergoing a palliative surgical procedure at our center prior to complete repair were excluded. We also excluded patients with extracardiac malformations requiring surgical interventions during the same cardiopulmonary bypass (CPB) as the cardiac surgery.

### 2.3 Procedure

Upon arrival at our center, we routinely performed a general and complete cardiological workup to confirm the ToF diagnosis and characterizing its specific anatomy. If needed, a complementary anatomic exam was performed (CT angiography or cardiac MRI).

Anesthesia [15], perioperative and postoperative management followed current international standards. Cell saver was routinely used to reduce the need for transfusion [16]. After CPB, the volemia was optimized with modified UltraFiltration (MUF) [17,18]. Thoracic and mediastinal drains were routinely positioned before thoracic closure.

### 2.4 Surgical technique repair

Under CPB, the VSD was closed with a patch, and the RVOTO enlarged. The specific surgical technique was tailored to the anatomy and tissue quality. We classified the surgical techniques to relieve RVOTO into three categories: patch-enlargement with preservation of the native pulmonary annulus (PVSR), patch-enlargement without preservation of the native pulmonary annulus (transannular patch, TP), and replacing the RV-PA connection (RV-PA) with a biological valved prosthesis (Contegra® or homograft). The PVSR was always the first surgical strategy attempt, and the TP or RV-PA were used as alternative techniques when a functional native pulmonary valve could not be preserved.

### 2.5 Data source

We retrospectively analyzed the medical file records of each identified patient (Soarian, Siemens® and MetaVisionSuite, iMDSoft®). Baseline demographic information, anatomic cardiac diagnosis, non-cardiac associated anomalies, preoperative factors, intraoperative and surgical items, postoperative factors, complications and mortality were collected and transcribed into a pre-defined spreadsheet. The postoperative complications were as described in the literature: death, need for ECMO support, surgical reintervention within 30-days, delayed chest closure, myocardial infarction, chylothorax, renal failure requiring renal replacement therapy, and neurological deficit.

A pediatric cardiologist measured the cardiac anatomic structures on the transthoracic echocardiograms (Xcelera, Philips®). As there are no RV free wall thickness Z-score in the literature and adequate correlation of the echocardiographic RV wall thickness measurement [19] with good reproducibility [20] is not demonstrated, we measured a ratio between RV anterior wall thickness in diastole (RVAWd) and left ventricle posterior wall thickness in diastole (LVPWd), expressing the relative right ventricular hypertrophy. For healthy adults, this ratio lies at approximately 0.3–0.34 [21].

### 2.6 Statistical analysis

The statistical analysis was performed with Stata 17.0 (StatCorp®). Continuous parameters are presented as median [interquartile range], and discrete parameters as absolute numbers (percentage). Continuous data were compared with the Wilcoxon-Mann-Whitney test, respectively with the Kruskal-Wallis test followed by the Dunn's post hoc procedure. Proportions were analyzed with the Chi-square test. Statistical significance was inferred at a value of p < 0.05. We analyzed the preoperative anatomy, perioperative and postoperative results depending on the three types of surgical technique repair. We performed a univariate and multivariate analysis to identify which parameters were independently predictive of PVSR.

## 3 Results

### 3.1 Patients

One hundred sixty-five patients met the inclusion criteria. Four of them were excluded from the perioperative and postoperative analysis because of extracardiac malformations (severe tracheal stenosis).

### 3.2 Demographic parameters

Baseline demographic and laboratory characteristics are reported in Table 1. The median age at repair was 4.5 [3.0–6.3] years with a male to female ratio of 1.75:1. Failure to thrive was observed in the majority of patients with a median WHO Z-score for weight, height, and BMI of −1.7 [−2.5 to −1.0], −1.4 [−2.4 to −0.7], and −1.3 [−2.3 to −0.3], respectively. All patients had a chronic cyanotic form of ToF with median peripheral pulse oximetry of 78% [70–85], increased haemoglobin level (163 g/L [142–190]) and hematocrit value (50.5% [44.0–59.1]).

**Table 1:** **-** Demographic parameters.

	Total	PVSR	RV-PA	TP	*p-value*
Number of patients	165	77 (46.7)	25 (15.1)	63 (38.2)	
Sex					*0.20*
Male	105 (63.6)	54 (51.4)	16 (15.3)	35 (33.3)	
Female	60 (36.4)	23 (38.3)	9 (15.1)	28 (46.6)	
Age at surgical repair (years)	4.5 [3.0–6.3]	4.6 [3.0–7.1]	4.6 [2.8–7.8]	4.1 [3.0–5.2]	*0.39*
Weight (kg)	13.5 [10.9–16.5]	14.0 [11.0–17.0]	13.5 [11.0–18.5]	13.2 [10.7–15.5]	*0.59*
Height (cm)	98 [88–111]	99 [90–112]	96 [89–114]	98 [87–107]	*0.72*
BMI (kg/m^2^)	14.0 [12.8–15.2]	14.1 [13.0–15.2]	14.1 [13.0–15.3]	13.5 [12.7–15.0]	*0.54*
Peripheral pulse oximetry (%)	78 [70–85]	80 [73–88]	75 [65–82]	77 [70–82]	***0.028***^***#***^
Hematocrit (%)	50.5 [44.0–59.1]	48.0 [41.7–57.0]	52.6 [47.4–64.5]	52.8 [47.5–59.1]	***0.015***^***#***^

Continuous variables are presented as median [IQR] and categorical variables as N (%). BMI: body mass index.

^#^p < 0.05 while comparing PVSR versus RV-PA and TP.

### 3.3 Preoperative echocardiographic parameters

Table 2 shows preoperative transthoracic echocardiographic anatomic and hemodynamic characteristics. Regarding the measurement of the RVOT, hypoplasia was the most relevant at the level of the main pulmonary artery and at the pulmonary valve annulus (Z-score of −3.41 [−4.93 to −2.00] and −1.84 [−3.42 to −0.85], respectively). The proximal pulmonary arteries were normal-sized in most patients: right pulmonary artery (RPA) Z-score −1.29 [−2.37 to 0.01] and left pulmonary artery (LPA) Z-score −0.43 [−1.82 to 0.78]. The RVAWd/LVPWd ratio quantified the relative severity of the right ventricle hypertrophy with a ratio of 1.21 [1.01 to 1.50].

**Table 2:** **-** Preoperative parameters.

Cardiac associated malformation	Total				
Interatrial shunt	42 (25.5)				
Aberrant coronary artery	5 (3.0)				
Anomaly of the systemic venous return	10 (6.1)				
Persistent ductus arteriosus	10 (6.1)				
Right aortic arch	17 (10.3)				
**Preoperative echocardiography**	**Total**	**PVSR**	**RV-PA**	**TP**	***p-value***
Maximal instantaneous RVOT gradient (mmHg)	77 [62–88]	70 [56–84]	83 [74–91]	79 [64–90]	***0.011***^***#***^
Pulmonary valve annulus (mm)	11 [9-13]	12 [10-14]	9 [8-10]	10 [8-12]	***0.0001***^***#***^
Pulmonary valve annulus Z-score	−1.8 [-3.4 to −0.8]	−1.2 [-2.6 to −0.2]	−2.7 [-4.2 to −1.8]	−2.4 [-3.7 to −1.0]	***0.0001***^***#***^
Main pulmonary artery (mm)	9 [7-11]	10 [8-12]	9 [7-12]	7 [5-9]	***0.0001***^***§***^
Main pulmonary artery Z-score	−3.4 [-4.9 to −2.0]	−2.7 [-3.9 to −1.6]	−2.8 [-4.8 to −1.9]	−4.5 [-6.3 to −3.1]	***0.0001***^***§***^
Left pulmonary artery (mm)	7.6 [5.7–9.4]	7.8 [5.9–9.8]	8.8 [6.3–12.0]	7.2 [5.1–8.5]	***0.007***^***§***^
Left pulmonary artery Z-score	−0.4 [-1.8 - 0.8]	−0.2 [-1.8 - 0.9]	0.1 [-1.3 - 1.8]	−0.7 [-2.3 - 0.3]	*0.05*
Right pulmonary artery (mm)	7.6 [6.2–9.3]	7.7 [6.4–9.2]	9.1 [7.2–12.1]	7.1 [5.7–8.6]	***0.004***^***+***^
Right pulmonary artery Z-score	−1.3 [-2.4 to −0.0]	−1.3 [-2.1 to −0.3]	−0.3 [-1.4 - 1.3]	−1.9 [-2.8 to −0.2]	***0.004***^***+***^
RVAWd/LVPWd ratio	1.21 [1.01–1.50]	1.13 [0.98–1.40]	1.24 [1.02–1.59]	1.30 [1.15–1.53]	***0.036 °***

Continuous variables are presented as median [IQR] and categorical variables as N (%). RVOT: right ventricle outflow tract, RVAWd: right ventricle anterior wall thickness in diastole, LVPWd: left ventricle posterior wall thickness in diastole. °p < 0.05 while comparing TP versus PVSR.

^#^p < 0.05 while comparing PVSR versus RV-PA and TP.

^§^p < 0.05 while comparing TP versus PVSR and RV-PA.

^+^p < 0.05 while comparing RV-PA versus PVSR and TP.

### 3.4 Intraoperative data

Table 3 shows the perioperative characteristics of the cohort. The median CPB time was 125 [105–151] min with a median aortic cross-clamp of 76 [62–91] min and mild hypothermia at 33.7 [32.1–34.8] °C. The exact anatomic description of the pulmonary valve and cusps was not systematically reported in the operative reports, with more than half of the data missing. The PVSR could be performed in 46.6% of patients, and only 11.2% had a modified Blalock-Taussig shunt done in their countries previous to the complete surgical repair. 98.8% of the patients underwent primary sternal closure.

**Table 3:** **-** Intraoperative data and postoperative outcomes.

Intraoperative data	Total	PVSR	RV-PA	TP	*p-value*
Number of patients	161	75 (46.6)	24 (14.9)	62 (38.5)	
Cardiopulmonary bypass time (min)	125 [105–151]	114 [94–135]	130 [113–146]	147 [118–173]	***0.0001***^***#***^
Aortic cross-clamp time (min)	76 [62–91]	73 [59–90]	82 [55–97]	79 [66–93]	*0.33*
Minimal hypothermia (°C)	33.7 [32.1–34.8]	34.1 [32.3–35.0]	33.0 [28.1–33.6]	33.9 [32.9–34.8]	***0.002***^***§***^
**Surgical technique**					
Preoperative modified BT shunt	18 (11.2)	7 (38.9)	6 (33.3)	5 (27.8)	***0.013***
Primary sternal closure	159 (98.8)	75 (100)	24 (100)	60 (96.8)	***-***
**ICU and Hospital parameters**					
Invasive ventilation time (hours)	26 [21–72]	25 [19–46]	25 [23–70]	49 [22–133]	***0.004***^***§***^
ICU length of stay (days)	5.9 [4.7–7.9]	5.2 [4.1–6.9]	5.1 [4.4–6.5]	7.0 [5.0–11.0]	***0.0008***^***§***^
Hospital length of stay (days)	9.0 [7.0–14.0]	8.0 [7.0–12.0]	9.0 [8.0–12.5]	11.5 [8.0–19.0]	***0.002***^***§***^

Continuous variables are presented as median [IQR] and categorical variables as N (%). ICU: intensive care unit, BT shunt: Blalock-Taussig shunt.

^#^p < 0.05 while comparing PVSR versus RV-PA and TP.

^§^p < 0.05 while comparing TP versus PVSR and RV-PA.

### 3.5 ICU and hospital lengths of stay

The median duration of invasive ventilation was 26.1 [21.3–71.7] hours after CPB weaning. The median ICU and hospital lengths of stay (LOS) were 5.9 [4.7–7.9] and 9.0 [7.0–14.0] days, respectively (Table 3).

### 3.6 Postoperative complications

There was no death in the 161 successive complete surgical repairs of ToF. The surgical reintervention rate at 30 days was 8.1% (N = 13) with an overrepresentation in the TP-group (N = 9/62, 14.5%) compared to PVSR-group (N = 4/75, 5.3%). There was no reintervention at 30 days in the RV-PA group. Three reinterventions were permanent pacemaker implantation (N = 2 in TP-group, N = 1 in PVSR-group). The residual cumulative postoperative complication rate was 4.3% (2 delayed chest closure, 1 ECMO, 3 chylothorax and 1 neurological deficit) with again overrepresentation in the TP-group (N = 6/62, 9.7%) compared to PVSR-group (N = 1/75, 1.3%).

### 3.7 Residual lesions at 30 days

At one month postoperative follow-up, the residual RVOTO was mild with a median maximal instantaneous RVOT gradient of 22 [14-32] mmHg. More than half of the patients (56.5%) had no or mild residual pulmonary regurgitation (Table 4).

**Table 4:** **–** Residual cardiac lesions at 30 postoperative days.

	Total	PVSR	RV-PA	TP	*p-value*
Residual maximal instantaneous RVOT gradient (mmHg)	22 [14-32]	21 [15-30]	19 [13-32]	25 [15–36]	*0.27*
**Residual pulmonary insufficiency**					
none	19 (11.8)	15 (78.9)	3 (15.8)	1 (5.3)	
mild	72 (44.7)	40 (55.6)	19 (26.4)	13 (18.0)	
moderate	43 (26.7)	17 (39.6)	1 (2.3)	25 (58.1)	
severe	27 (16.8)	3 (11.1)	1 (3.7)	23 (85.2)	*0.65*

Continuous variables are presented as median [IQR] and categorical variables as N (%). RVOT: right ventricle outflow tract.

### 3.8 Demographic and preoperative echocardiographic findings grouped by type of surgical repair

In the secondary analysis comparing the 3 different groups, we observed no significant differences in the demographic characteristics. As shown in Table 1, Table 2, the PVSR-group had a milder form of ToF compared to RV-PA and TP in term of significantly higher peripheral pulse oximetry (80% [73–88] vs 75% [65–82] vs 77% [70–82] respectively, p = 0.0280), lower maximal instantaneous RVOT gradient (70 [56–84] vs 83 [74–91] vs 79 [64–90] mmHg, p = 0.0112) and a lower RVAWd/LVPWd ratio (1.13 [0.98–1.40] vs 1.24 [1.02–1.59] vs 1.30 [1.15–1.53], p = 0.0360).

**Fig. 1:**
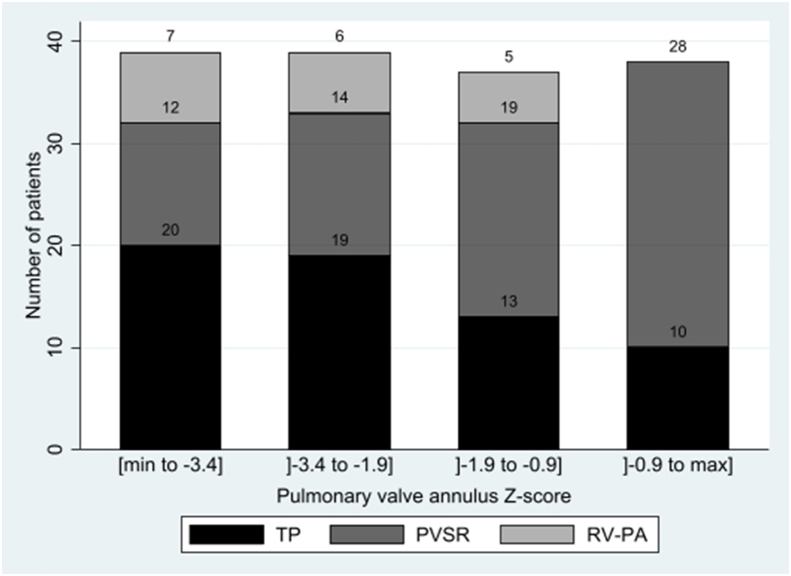
Number of patients for each surgical technique repair depending on the pulmonary valve annulus Z-score.

Hypoplasia was more relevant at the level of the main pulmonary artery and the pulmonary valve annulus level, with a significantly larger annulus in the PVSR-group (p = 0.0001) and a significantly smaller main pulmonary artery in the TP-group (p = 0.0001) (Table 2). Fig. 1 shows the different types of surgical repair distribution conditional to the pulmonary valve annulus Z-score.

### 3.9 Perioperative and postoperative outcomes by type of surgical repair

We detected a significantly shorter invasive ventilation time (25 [19–46] vs 25 [23–70] vs 49 [22–133] hours, p = 0.0043), ICU LOS (5.2 [4.1–6.9] vs 5.1 [4.4–6.5] vs 7.0 [5.0–11.0] days, p = 0.0008) and hospital LOS (8.0 [7.0–12.0] vs 9.0 [8.0–12.5] vs 11.5 [8.0–19.0] days, p = 0.0024) for patients with preserved pulmonary valve function (PVSR and RV-PA groups) as compared to TP-group (Table 3). Because TP and RV-PA connection replacement were alternatives undertaken after failure to realize a pulmonary valve-sparing repair, we observed a significantly longer median CPB time in both groups (147 [118–173], 130 [113–146] versus 114 [94–135] min, respectively, p = 0.0001).

### 3.10 Complications and outcomes at 30 days by type of surgical repair

There was a significantly higher reintervention rate in the TP-group (N = 9/62) versus preserved pulmonary valve function (PVSR and RV-PA groups, N = 4/99), p = 0.0176.

Due to the humanitarian setup, a follow-up in Switzerland could only be accomplished until 30 days after the surgery. At this timepoint, the residual maximal instantaneous gradient over the RVOT was mild without differences between the three groups (22 [14-32] vs 21[15-30] vs 19 [13-32] mmHg, p = 0.27), and, as expected, the severity of residual pulmonary valve regurgitation was less in the PVSR and RV-PA-groups than in the TP-group (Table 4).

### 3.11 Univariate and multivariate models identifying parameters predictive for PVSR

Univariate analysis (Table 5) identified multiple parameters associated with the failure to proceed with a pulmonary valve-sparing repair: smaller pulmonary valve annulus, hypoplasia of the main pulmonary artery (Z-score inferior to −3.4), severe RVOTO (gradient over 77 mmHg), and elevated RVAWd/LVPWd ratio (>1.2).

**Table 5:** **–** Univariate regression analysis: Pulmonary valve-sparing repair.

	OR (95% CI)
Pulmonary valve annulus Z-score < −3.0	2.20 (1.06–4.55)
Pulmonary valve annulus Z-score < −4.0	3.24 (1.21–8.69)
Main pulmonary artery Z-score < −3.4	3.42 (1.76–6.64)
RVOTO >77 mmHg	1.95 (1.01–3.76)
RVAWd/LVPWd ratio >1.2	2.45 (1.25–4.80)

RVOTO: Right ventricle outflow tract obstruction, RVAWd: right ventricle anterior wall thickness in diastole, LVPWd: left ventricle posterior wall thickness in diastole.

Interestingly, multivariate analysis eliminated the pulmonary valve annulus Z-score as an independent parameter predicting the ability to preserve pulmonary valve annulus integrity in the studied population. However, multivariate analysis (Table 6) identified severe right ventricular hypertrophy (RVAWd/LVPWd ratio >1.2), severe RVOTO (gradient over 77 mmHg) and hypoplasia of the main pulmonary artery (Z-score inferior to −3.4) as significant independent predictors for failure to preserve the pulmonary annulus at surgery.

**Table 6:** **–** Multivariate regression analysis: Pulmonary valve-sparing repair.

	OR (95% CI)
Main pulmonary artery Z-score < −3.4	3.00 (1.44–6.27)
RVOTO >77 mmHg	2.29 (1.07–4.93)
RVAWd/LVPWd ratio >1.2	2.67 (1.24–5.75)

RVOTO: Right ventricle outflow tract obstruction, RVAWd: right ventricle anterior wall thickness in diastole, LVPWd: left ventricle posterior wall thickness in diastole.

## 4 Discussion

Humanitarian patients with late complete surgical repair of ToF are an unusual population in Western Countries. Furthermore, they represent many patients worldwide in developing countries where pediatric cardiac surgical programs have started. This study aimed to analyze the feasibility of applying current strategies for ToF surgical repair to this population. To the best of our knowledge, this is the largest report of late complete surgical repair of ToF in Western Countries.

### 4.1 Surgery

In this study, no mortality was encountered among the 165 successive surgeries realized over 13 years, which is similar to the results obtained for timely ToF repair [6].

A cut-off value of pulmonary valve annulus Z-score of less than −3 to −4 is often reported as a technical threshold to realize a PVSR [8]. In comparison, in the current study, we had a 47% rate of PVSR, and the possibility of preserving the pulmonary valve annulus was not linked with the pulmonary valve annulus Z-score in multivariate analysis.(Fig. 1).

Of note, the valvular tissue can adapt to environmental conditions, mainly mechanical loading [22]. Therefore, we can hypothesize that chronic turbulent flow induces an extended remodeling of the valve leaflets by increased shear stress, leading to decreased tissue elasticity and plasticity [23]. These factors could explain the limited surgical possibilities to maintain postoperative functional valve leaflets. As the anatomy of the pulmonary valve and tissue quality description of the leaflets were not systematically available in the surgical report, we could only suppose that it could be an explanation for the lower PVSR rate in our population.

Another group [24] describing late correction of tetralogy of Fallot experience reported a 96% rate of PVSR with a median pulmonary valve Z-score of −1.9±1.6. The difference in PVSR rate between the two studies might be related to a higher baseline saturation and absence of preoperative BT shunt in all patients suggesting a less severe form of ToF in this latter study.

### 4.2 Complications, ICU and hospital lengths of stay

The median invasive ventilation time, ICU and hospital LOS were equivalent to contemporary reports of other centers [[24], [25], [26]]. We observed a significant difference in the preoperative echocardiography parameters between the PVSR-group compared to TP- and RV-PA-group with lower pulmonary annulus Z-score, lower peripheral pulse oximetry saturation, and a higher RVAWd/LVPWd ratio reflecting more RV hypertrophy in these two latter groups. Severe forms of ToF are associated with restrictive physiology of the RV [27], which is linked with postoperative low cardiac output syndrome and prolonged ICU and hospital LOS [28,29].

Finally, in this study, the RV-PA and the TP-groups shared the same preoperative characteristics, the same setup for restrictive physiology of the RV, identical intraoperative data, and residual RVOTO. However, they significantly differed with regard to invasive ventilation time, ICU and hospital LOS, suggesting that, in this population, the preservation of pulmonary valve function is a crucial parameter in the postoperative short-term period, although we meanwhile know that, over the long term, pulmonary regurgitation is pretty well tolerated in the presence of restrictive RV physiology [30,31].

Surprisingly, contrary to the literature on ToF surgical repair in infants, in multivariate analysis, we observed that the magnitude of pulmonary valve annulus hypoplasia was not an independent factor predicting the failure to preserve the integrity of the pulmonary annulus. Probably, in long-lasting ToF, the dimension of the pulmonary annulus just represents part of the changes in the RV restrictive physiology, and pulmonary valve annulus size should therefore not be considered the unique parameter to decide on the best surgical approach.

### 4.3 Limitations of the study

The included population has an intrinsic selection bias, representing exclusively the children who could be diagnosed with ToF in their native countries and survive long enough to be transferred to Switzerland for surgical repair. However, as per their baseline characteristics, we found similarly associated lesions and a male overrepresentation as described in the general literature on ToF [32]. The phenotype of some children was suspicious for associated genetic abnormalities. Owing to the humanitarian setup, however, we were unable to perform genetic testing, thereby precluding the evaluation of genetic syndrome's influence on the outcomes [33]. Finally, pulsed doppler echocardiography demonstrating forward diastolic flow in the pulmonary artery as a marker of restrictive physiology of the RV would have been an exciting parameter to be measured and compared to stratify the population's study better but, due to the retrospective design, was incompletely collected and could therefore not be assessed.

## 5 Conclusion

This study delivers three main results. First, late complete surgical repair of ToF can successfully be performed with low mortality and morbidity. Second, endorsing, similar to newborns and infants, a pulmonary valve-sparing repair based only on the dimension of the pulmonary valve annulus seems not to be adequate for these patients. The best surgical option for the best short and long-term outcome has to be further evaluated, and it is probably based on a multimodal approach where the size of the pulmonary vessels, valve, as well as the degree of RVOTO and RV hypertrophy are considered. Finally, in this category of patients with ToF, the preservation of the pulmonary valve function (pulmonary valve-sparing repair or replacement of the RV-PA connection with a biological valved prosthesis) is associated with a shorter invasive ventilation time, ICU and hospital LOS.

## Declaration of competing interest

The authors declare that they have no known competing financial interests or personal relationships that could have appeared to influence the work reported in this paper.
